# Recurrent purpura due to alcohol-related Schamberg’s disease and its association with serum immunoglobulins: a longitudinal observation of a heavy drinker

**DOI:** 10.1186/s13256-016-1065-6

**Published:** 2016-10-31

**Authors:** Udo Bonnet, Claudia Selle, Katrin Isbruch, Katrin Isbruch

**Affiliations:** 1 Department of Psychiatry, Psychotherapy and Psychosomatic Medicine, Evangelisches Krankenhaus Castrop-Rauxel, Academic Teaching Hospital of the University of Duisburg/Essen, Grutholzallee 21, 44577 Castrop-Rauxel, Germany; 2 Department of Psychiatry and Psychotherapy, University of Duisburg/Essen, Virchowstr. 174, 45147 Essen, Germany

**Keywords:** Purpura, Schamberg’s disease, Alcohol, Acetate, Hypersensitivity-like reaction

## Abstract

**Background:**

It is unusual for purpura to emerge as a result of drinking alcohol. Such a peculiarity was observed in a 55-year-old man with a 30-year history of heavy alcohol use.

**Case presentation:**

The Caucasian patient was studied for 11 years during several detoxification treatments. During the last 2 years of that period, purpuric rashes were newly observed. The asymptomatic purpura was limited to both lower limbs, self-limiting with abstinence, and reoccurring swiftly with alcohol relapse. This sequence was observed six times, suggesting a causative role of alcohol or its metabolites. A skin biopsy revealed histological features of purpura pigmentosa progressiva (termed *Schamberg’s disease*). Additionally, alcoholic fatty liver disease markedly elevated serum immunoglobulins (immunoglobulin A and immunoglobulin E), activated T-lymphocytes, and increased C-reactive protein. In addition, moderate combined (cellular and humoral) immunodeficiency was found. Unlike the patient’s immunoglobulin A level, his serum immunoglobulin E level fell in the first days of abstinence, which corresponded to the time of purpura decline. Systemic vasculitis and clotting disorders were excluded. The benign character of the purpura was supported by missing circulating immune complexes or complement activation. An alcohol provocation test with vinegar was followed by the development of fresh “cayenne pepper” spots characteristic of Schamberg’s disease.

**Conclusions:**

This case report demonstrates that Schamberg’s disease can be strongly related to alcohol intake, in our patient most likely as a late complication of severe alcoholism with alcoholic liver disease. Immunologic disturbances thereby acquired could have constituted a basis for a hypersensitivity-like reaction after ingestion of alcohol. Schamberg’s disease induction by vinegar may point to an involvement of acetate, a metabolite of ethanol.

## Background

Cutaneous manifestations of alcohol abuse can rarely include purpura, which at first sight could be related to acquired clotting defects such as thrombocytopenia and vascular fragility resulting from liver cirrhosis [1, 2]. Purpura is caused by hemorrhage of small vessels in the skin or mucous membranes and emerges as red or purple spots that do not blanch upon application of pressure on the lesion’s surface [3]. Usually, these skin lesions are a hallmark of coagulation disorders or vasculitis [3], such as disseminated intravascular coagulation, idiopathic thrombocytopenic purpura, Henoch-Schönlein purpura (HSP), or hypersensitivity (leukocytoclastic) vasculitis [3–7].

There are a few case reports in the literature of purpura associated with alcohol use. Among them, three were related to HSP [8–10], one was associated with Sjögren’s syndrome [11], another one appeared simultaneously with an aspirin-induced platelet dysfunction [12], and a further one was suggested to result from thrombotic thrombocytopenic purpura due to alcohol binge drinking [13].

Moreover, pigmented purpuric dermatoses, such as purpura pigmentosa progressiva (termed *Schamberg’s disease* [SD]), have been described as being associated with chronic drinking [14]. SD can occur in every age group from childhood to senium, is usually benign and asymptomatic, and appears preferentially on the lower limbs. The course is usually persistent and chronic [15], with the exception of drug-induced transients, considering inflammatory purpura under the influence of nonsteroidal anti-inflammatory drugs, acetaminophen, diuretics, meprobamate, ampicillin, and amlodipine [16–18]. Histologically, SD is characterized by patchy parakeratosis, mild spongiosis of the stratum malpighii, and lymphoid capillaritis. Monocytes, histiocytes, lymphocytes, and occasionally mast cells infiltrate the perivascular area. Endothelial cells swell and proliferate, thus forming a rhexis of capillary walls promoting diapedesis of erythrocytes and subsequent deposition of hemosiderin [16, 19]. SD’s etiology is obscure, but antigen and other cellular immune mechanisms appear to be involved [19–21]. In addition, an abnormal, spasmlike motility of the capillaries, probably via dysautonomia [22] and stasis pigmentation [23], have been described as being associated with SD.

In this case report, the close interaction of severe alcohol abuse and occurrence of SD is demonstrated. For the first time, to our knowledge, an association of purpura with serum levels of immunoglobulin E (IgE), as well as the induction of fresh SD purpura via drinking vinegar, is shown.

## Case presentation

An adult Caucasian alcohol-dependent patient who developed purpura after drinking alcohol was clinically observed from May 2005 to April 2016 at our institution (Table 1). The last 2 years (“purpuric phase”; cf. Table 1) were prospectively studied, and the time before that (“nonpurpuric phase”) was studied retrospectively. In the “purpuric phase,” a skin biopsy was performed, and routine diagnostics at every detoxification treatment were expanded by determination of serum immunoglobulins, complement factors C3 and C4, and circulating immune complexes. Additionally, abdominal ultrasound as well as cerebral and internal diagnostics for clotting defects, collagenoses, and systemic vasculitis were performed. A provocation test with vinegar was performed, assumed to be a challenge test for acetate, one main metabolite of ethanol [24]. For ethical, therapeutic, and toxicological reasons, provocation tests using ethanol itself or acetaldehyde were not performed.

**Table 1:**
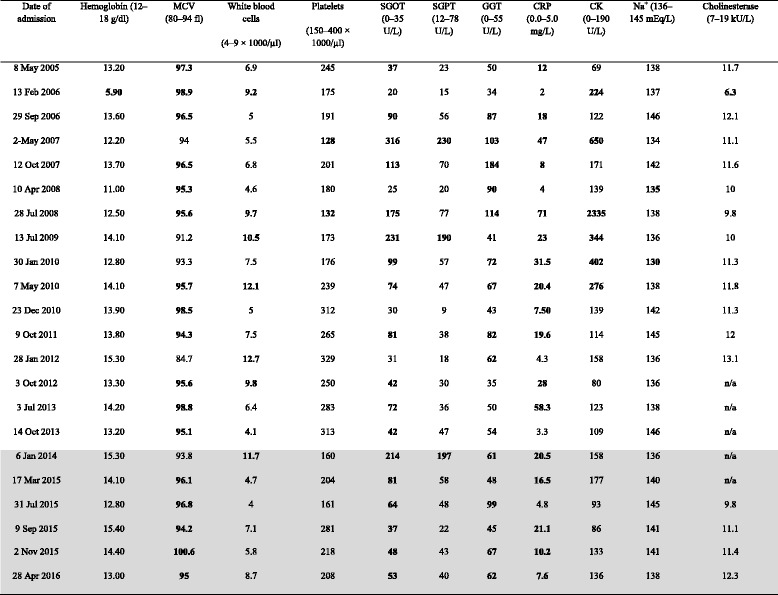
Routine laboratory results at the patient’s multiple admissions

*Abbreviations: MCV* Mean corpuscular volume, *SGOT* Serum glutamic oxaloacetic transaminase, *SGPT* Serum glutamate-pyruvate transaminase, *GGT* γ-Glutamyltransferase, *CRP* C-reactive protein, *CK* Creatine kinase, *n/a* Not applicable

Admissions with purpuric rashes are shaded (“purpuric phase”). Reference values are given in boldface type. Pathological laboratory results revealed slightly elevated uric acid in most measurements (not shown here)

### Principal symptoms

**Fig. 1:**
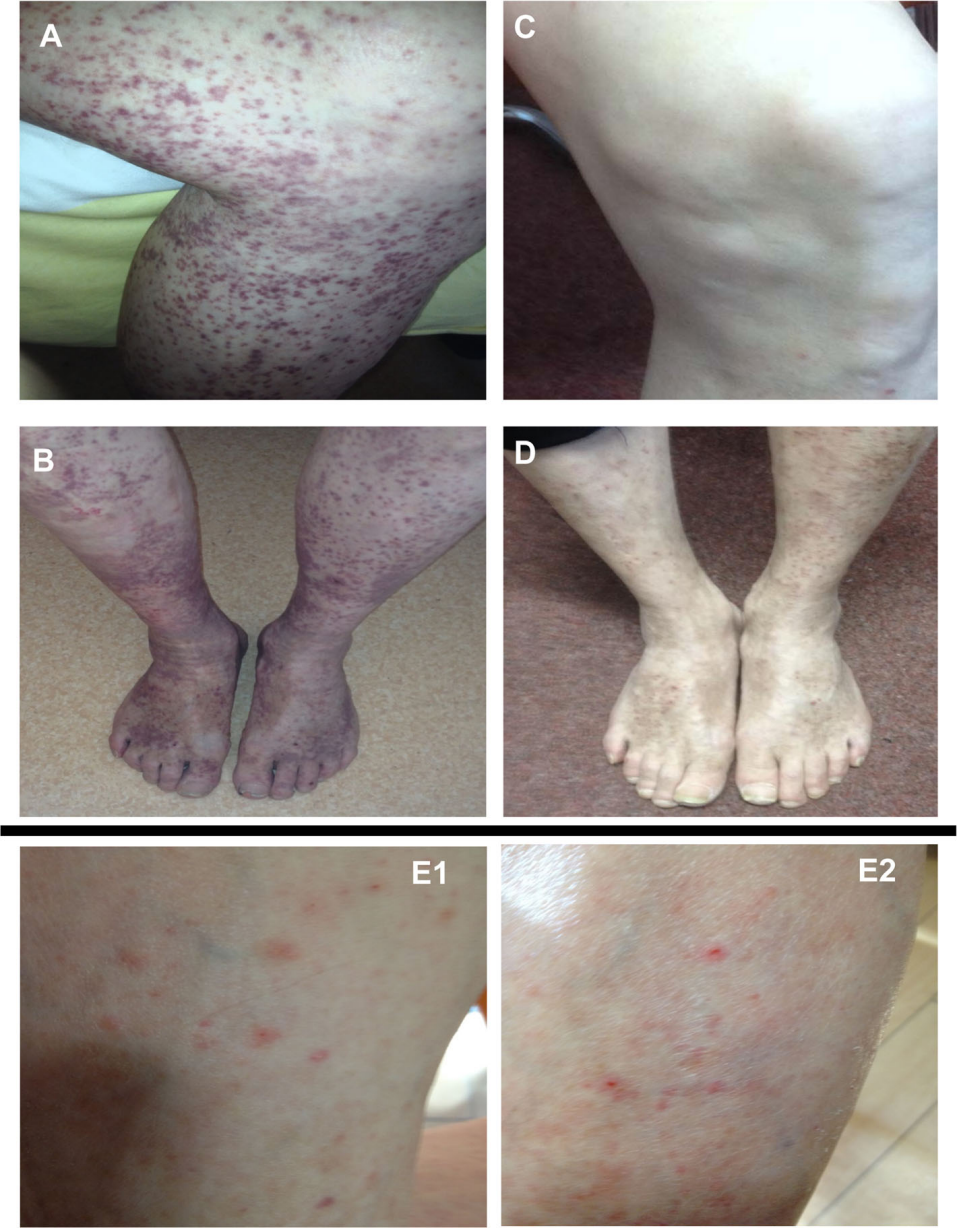
Purpuric rashes at admission (**a, b**) and blanched lesions with confluent hyperpigmentation after 14 days of controlled alcohol abstinence (**c, d**) in November 2015. Fresh purpura (“cayenne pepper” spots) after 24 h of drinking vinegar (50 ml four times daily) (**e1** left tibia, **e2** right tibia) in April 2016 at the end of a 10-day inpatient detoxification treatment

During the last 2 years that we followed him at our clinic, a German-speaking white man of Polish descent (55 years old, 166 cm in height, 62 kg in weight, single, unemployed, and living in his own apartment) with a severe alcohol use disorder had six inpatient detoxification treatments (Table 1). Upon each admission, his physical examinations revealed purpuric rashes appearing symmetrically on his lower extremities that may have followed gravity (Fig. 1a, b). The patient reported that the purpura had emerged during periods of excessive drinking of vodka or beer (usually >300 g/day of ethanol), began to resolve in the first week of abstinence, and erupted again after relapsing alcohol use. The skin rashes were not associated with itching, pain, or functional restrictions in his lower limbs. He reported no fever, nausea, vomiting, migrating arthralgia, or abdominal pain in association with these rashes. His oral mucosa and other skin areas were not affected. In the years before presentation, he had never noticed such skin lesions, which were confirmed by reviewing his chart records in our hospital (Table 1). Neither allergic reactions nor flushes were previously or currently noticed. He had no personal or family history of atopy, asthma, or purpura and denied using medications or drugs. The remaining examination showed cerebellar ataxia, affective lability, difficulties with concentration, and impaired memory, all declining with detoxification. Mild executive dysfunction and slight signs of a sensorimotor polyneuropathy on his lower extremities, as well as an enlarged liver, persisted after the detoxifications. At the end of the benzodiazepine-mediated withdrawal treatments, which lasted 7–21 days, the patient’s purpura always began to blanch, leaving confluent patches of hyperpigmentation behind (Fig. 1d), which is characteristic of SD [19–21].

### Medical and addiction history

The patient reported drinking alcohol nearly daily since he was 17 years old. In the past 11 years, he had completed multiple alcohol detoxification treatments in our ward (Table 1). Several neuropsychiatric and physical sequelae of heavy alcohol use had been diagnosed, such as alcoholic fatty liver disease (AFLD), withdrawal seizures and deliria, polyneuropathy, cerebral and cerebellar atrophy, and personality change. The patient reported no further diseases in his history. He drank preferentially large amounts of vodka and beer containing 200–500 g/day of ethanol and usually was brought to the hospital by ambulance or caregivers, when he was found helplessly drunk in the streets or at home. Upon admission, his blood alcohol concentrations were between 0.25 % and 0.42 %. He had smoked usually up to 30 cigarettes per day since his early adolescence.

### Diagnostic analyses

**Fig. 2:**
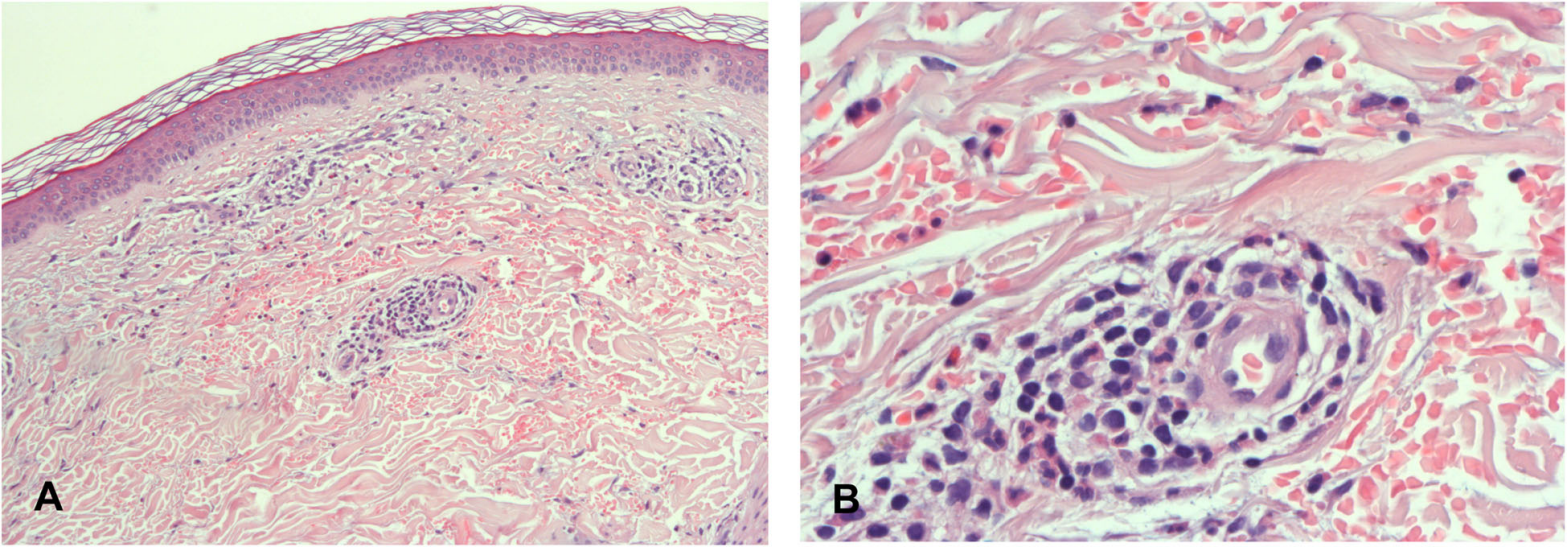
Histology of the skin biopsy. **a** There is a light perivascular lymphocytic infiltration in the upper dermis without involvement of the epidermis. **b** The blood vessels show endothelial reaction without destruction. An extravasation of red blood cells can be seen (courtesy of Prof. Dr. Kasper, MD, Institut für Pathologie am Clemenshospital, Münster, Germany)

Routine blood analysis, including cystatin C, ammonium, and ferritin, were largely normal, except for moderately increased transaminases and C-reactive protein (CRP), over the years (Table 1). On his abdominal ultrasound, signs of AFLD were found, such as enlarged liver combined with increased echogenicity and coarsened echotexture. Serologic tests for hepatitis, HIV, and lues were negative. Current electrophysiology revealed mild neuropathy in his lower limbs. Regarding the hemorrhagic purpura, an underlying bleeding disease or thrombocytopenia (Table 1) could be reliably excluded. Also, no evidence of internal or cerebral involvements of vasculitis was found via repeated neuroimaging (brain computed tomography [CT] and magnetic resonance imaging [MRI] including angiography), electrocardiograms, angiology, and phlebologic diagnostics, and thoracic as well as abdominal CT scans, during the detoxification treatments in the “purpuric phase.” Capillary microscopy of the feet revealed no signs of vasculitis. This was supported by a skin biopsy of a small purpuric area that revealed characteristic histological features of SD; that is, a perivascular lymphocytic infiltrate associated with reactive endothelial changes and extravasated red blood cells. The vessel walls were intact, and fibrin and thrombi are lacking (Fig. 2). The patient’s drinking vinegar was followed by fresh “cayenne pepper” spots (Fig. 1e1, e2) characteristic of SD [19–21]. These appeared not immediately but 24 h after the patient ingested the vinegar.

Immunodiagnostics comprised the determination of serum immunoglobulin levels, autoantibodies, circulating immune complexes, haptoglobin, hemopexin, cryoglobulins, and complement components C3 and C4, as well as serum protein electrophoresis, lymphocyte typing, immunofixation, and Coombs tests. We found markedly increased serum levels of IgE and IgA (IgA1 and IgA2) (Table 2). Unlike IgA, the patient’s IgE levels decreased with detoxification (Table 2). Lymphocyte typing revealed lowered B-lymphocytes and CD4 T-cells (at average CD4/CD8 ratio, natural killer cells, and cytotoxic cells) (cf. Table 2), pointing to a moderate combined (humoral and cellular) immune deficiency, which is typical for individuals with severe alcoholism [25]. In addition, a specific response of the cellular immune system (elevated ratio of activated T-lymphocytes) was found (cf. Table 2), which may point to type IV hypersensitivity. The patient’s results were repeatedly unremarkable for serum protein electrophoresis, troponin, and Coombs tests; haptoglobin, hemopexin, cryoglobulins, IgM, IgG1, and IgG4levels; and complement (C3 and C4) and immunofixation tests. Moreover, we found no circulating immune complexes (C1q-IgG and C3d-IgG; evaluated with C1q- and C3d-binding assays) or autoantibodies (antinuclear antibodies, extractable nuclear antigens, antineutrophil cytoplasmic antibodies, thyroid antibodies, and liver-specific or anti-histone antibodies).

**Table 2:**
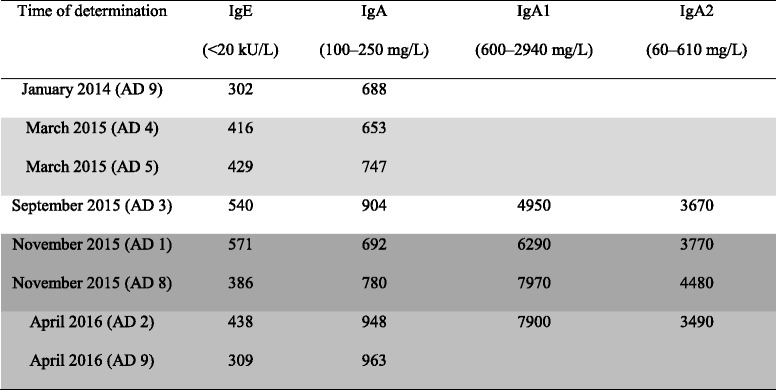
Immunodiagnostics in serum

The patient’s immunoglobulin (IgA) level appeared to increase, while his IgE levels fell, in tandem with abstinence days (AD). Reference levels are given in brackets, and measurements in variable intervals during the same inpatient treatment are shaded. Not shown in this table is the lymphocyte typing done in September 2015 with the following results: CD19 B-lymphocytes (132–422/μl) 92/μl, CD4 T-cells (645–1289/μl) 612/μl, and activated T-lymphocytes (43–270/μl) 303/μl with a ratio (3.0–12,5 %) of 23.4 %. The immunological results point to a moderate combined cellular immunodeficiency (low CD19 B-lymphocytes, low CD4 T-cells at normal CD4/CD8 ratio) and a hypersensitivity-like reaction (elevated IgA and IgE with specific response of the cellular immune system [elevated ratio of activated T-lymphocytes])

Serum vitamin tests showed normal values of folic acid and methylmalonic acid, holotranscobalamin, thiamine, and pyridoxine and a slightly lowered level of calciferol, which was substituted. No evidence of proteinuria, hematuria, or drug abuse was found over the years the patient was followed at our clinic. Also, no blood was observed in several stool tests. At two admissions, the patient’s N-terminal prohormone of brain natriuretic peptide was slightly elevated and normalized during detoxification. The last two detoxification treatments were initially associated with generalized slowing in electroencephalograms and impairments of delayed recall and visuospatial skills according to the Montreal Cognitive Assessment, all of which normalized up to discharge. The patient’s Mini Mental State Examination score was unremarkable at that time (29 of 30 possible points). Actually, the patient’s abdominal CT scan and his normal serum cholinesterase (Table 1), prothrombin time, international normalized ratio, bilirubin, IgG, IgM, albumin, and protein electrophoresis laboratory results did not point to liver cirrhosis, which could have fostered the elevated serum immunoglobulins and combined immunodeficiency [25–27]. Nonetheless, even hardly affected liver function tests do not exclude an inflammatory progression of AFLD to advanced liver fibrosis [28, 29], which is gradually associated with these immune system anomalies, too [25, 27]. A liver biopsy was not performed.

### Course of the addiction treatment

During his multiple detoxification therapies (Table 1), the patient usually developed pronounced alcohol withdrawal symptoms that were treated with benzodiazepines, and he received thiamine supplementation every time. In the patient’s several subsequent inpatient rehabilitation programs, he had not achieved sustainable abstinence. The number of these treatments was negatively correlated with the duration of his abstinence periods, finally lasting less than 1 month after treatment. Therefore, the patient was taken closer to the hospital’s ambulatory addiction treatment program. In this setting, his abstinence periods lasted between 1 and 3 months. Comorbid affective or psychotic disorders could be excluded, and various anticraving medications were not effective. Disulfiram was associated with delirium and seizures in his history and therefore was not applied.

## Discussion

This longitudinal observation demonstrates the recurrence of purpura dependent on the intake of large amounts of alcohol, which happened after about 30 years of chronic, heavy drinking. Differential diagnoses, such as coagulopathies and vasculitis disorders, were excluded, whereas typical features of SD were found in a skin biopsy. Immunodiagnostics revealed markedly elevated IgE and IgA levels and activated T-lymphocytes. Symptoms of systemic IgA-mediated vasculitis (HSP), such as migrating arthralgia, gastrointestinal symptoms, renal dysfunction, proteinuria, or hematuria [30, 31], were not present. Furthermore, brain MRI revealed no cerebral micro- or macroangiopathy or hemorrhagic lesions [32].

### Alcohol, liver, and IgA

Acute alcohol binge drinking was recently shown to stimulate rapidly the humoral immune defense [33], and the authors of previous reports stated that chronic alcohol use is associated with an increase in total serum IgA with or without liver disease [27]. The degree of liver injury is strongly correlated with serum IgA activity [25, 27, 34], most likely due to weakened IgA clearance in the liver [26]. The majority of basal serum IgA is synthesized in the bone marrow, being the largest lymphoid tissue in the body. Another big portion of IgA is secreted from the mucosa, mainly from the intestinal mucosa [26]. It is assumed that IgA supports the oral tolerance of harmless antigens, such as nonreplicating proteins or food proteins, that have leaked into the circulation from the skin, respiratory system, or gastrointestinal tract [26]. At this juncture, the disturbed gut wall integrity of chronic drinkers [35] weakens the barrier against penetrating pathogens. This may play a central role in the stimulation of large IgA secretion into the circulation, which might contribute to inflammatory, destructive processes in alcoholic liver disease [35] that can further potentiate serum IgA activity due to progressing liver injury. IgA in patients with alcoholic liver disease may circulate as part of immune complexes [35], and IgA was found to deposit in the skin and kidneys in patients with alcoholic liver disease without purpura [35]. Notably, internal involvement could be possible on the basis of a case report in which authors described an association of progressive IgA-mediated nephropathy and liver cirrhosis [36].

The disturbed gut wall integrity could be responsible for the CRP elevations consistently found in heavy drinkers (cf. Table 1) [37]. CRP is recognized to be an inflammatory acute-phase protein, remarkably of hepatic origin.

### Alcohol, liver, and IgE

Allergic reactions to pure alcohol or its metabolites [38] are rare and mainly attributed to IgE-mediated urticaria or anaphylaxis [39, 40]. Unlike IgA, serum IgE has not been clearly associated with the severity of liver dysfunction [39]. Levels of IgE have been found to be increased and decreased alongside alcohol consumption and abstinence, respectively [39] (cf. Table 2). An alcohol-induced increase of IgE in the serum might modify the risk for IgE-mediated allergic sensitization. However, the clinical relevance has been estimated to be moderate [39]. An association of serum IgE elevations and the development of purpura and renal injury has been described in children with HSP [41, 42].

### Alcohol, liver, and cellular immune system

An unfavorable interaction between heavy alcohol use and liver injury (as described for the serum IgA activity) has been shown for the cellular immune system, too [25]. Chronic alcohol consumption affected both the number and functioning of circulating CD4 T-cells, and the more these were affected, the more the liver was damaged [25]. Moreover, the number of activated CD4 T-cells was described to be increased in individuals with chronic alcoholism [25], as in our patient (cf. legend of Table 2).

### Mechanism of the presented alcohol-related purpura

The mechanism of SD is largely unknown. Adaptations in cell-mediated immunity contribute to the genesis of this usually benign condition, deemed to be a capillaritis by most but not all authors [19–21]. SD affects capillaries close to the surface of the skin, predominantly in the lower limbs, allowing red blood cells to migrate into the skin [19–21]. This localization appears to resemble that of IgA-mediated vasculitis, which is characterized by deposition of IgA-containing circulating immune complexes within and around cutaneous capillaries [30, 43], and especially around postcapillary venules (typically leukocytoclastic vasculitis in histology) [44]. Intriguingly, SD was associated with markedly elevated serum IgA levels in our patient, too; nevertheless, circulating immunoglobulin complexes or complement activation [45] was not found, confirming the benign character of SD. Our patient’s purpura declined alongside detoxification, while his IgA levels persisted or increased (Table 2). Thus, at first sight, IgA levels might be merely “bystanders” of chronic alcohol drinking [27] rather than being directly involved in the pathogenesis of SD-related purpura. We are not aware of literature describing a progression from SD to systemic IgA-mediated vasculitis or an interaction of both diseases. However, IgA might have facilitated the fragility of cutaneous capillaries in our patient’s lower limbs, perhaps following gravity.

Mast cells were described to be involved in SD etiology [16, 19], but a timely correlation with IgE levels, as seen in our patient, has not been described before. One might speculate that the alcohol-mediated increase in IgE activity serves as a “spark” igniting SD purpura, which is usually chronic [15] but can be transient if it emerges after the administration of certain drugs, such as alcohol [14]. In our patient, the causative role of alcohol was underlined by the close temporal relationship between the occurrence of SD purpura and alcohol relapse.

It is worth noting that fresh purpura emerged after our patient drank vinegar, assumed to be a challenge test for the consumption of acetate, a metabolite of ethanol [24]. A similar observation was made in one previous case report of purpura due to alcohol consumption, notably with deposits of IgA in the dermal vessels but no histological signs of vasculitis in a biopsy of the purpuric macules [8]. Administration of 1 ml of 5 % acetic acid, however, was not followed by purpura [8], suggesting that technically negligible amounts of ethanol in vinegar may be “purpurogenic” or that this small amount of pure acetic acid was simply insufficient to “kindle” purpura. Over our patient’s years of heavy drinking, acute effects of alcohol or its metabolites on capillaries (possibly sustainably affected by IgA) involving IgE [8, 39, 46] or not involving IgE [22, 23, 47, 48] might have sensitized and eventually boosted the manifestation of purpura. We favor an IgE-mediated increase in the permeability of cutaneous capillaries [46, 48] facilitating the penetration of erythrocytes into the skin, thereby forming fresh purpura. We were reluctant to use the term *type 1 hypersensitivity* because the purpura did not develop immediately after the ingestion of alcohol or vinegar and skin-prick tests for ethanol or its metabolites were not performed. The patient had further changes of the immune system, such as activated T-lymphocytes, that may point to an additional delayed immune mechanism. Moreover, he had a negative history of previous allergic reactions. An individual genetic makeup might have predisposed the patient to SD, which was breaking through after further adaptations of the immune system due to several years of heavy drinking, most likely involving immunologic reactions of alcoholic liver damage and leakage of the gut wall barrier. In addition, dysautonomia [22] as well as ethanol- and/or stress-mediated changes in local skin immunology [25, 49] might be factors promoting the patient’s alcohol-related SD.

## Conclusions

This case report shows that alcohol-related purpura can be benign, despite occurring for the first time after many years of frequent and heavy alcohol use, and is not inescapably related to acquired or activated vasculitis or clotting disorders. Our patient developed a hypersensitivity-like reaction to ethanol and/or acetate (as shown by raised serum levels of IgE and IgA as well as activated T-lymphocytes) manifesting as SD with recurrent purpura as a consequence of chronic and heavy drinking. SD purpura should be considered in the list of dermatologic alcohol use disorders.

## Abbreviations

AD: Abstinence days
AFLD: Alcoholic fatty liver disease
C3 and C4: Complement factors
CD4 and CD8: Cluster of differentiation 4 and 8, respectively
CK: Creatine kinase
CT: Computed tomography
CRP: C-reactive protein
GGT: γ-Glutamyltransferase
HSP: Henoch-Schönlein purpura
Ig: Immunoglobulin
MCV: Mean corpuscular volume
MRI: Magnetic resonance imaging
SD: Schamberg’s disease
SGOT: Serum glutamic oxaloacetic transaminase
SGPT: Serum glutamate-pyruvate transaminase
