# Erratum to: Recurrent purpura due to alcohol-related Schamberg’s disease and its association with serum immunoglobulins: a longitudinal observation of a heavy drinker

**DOI:** 10.1186/s13256-016-1139-5

**Published:** 2016-11-23

**Authors:** Udo Bonnet, Claudia Selle, Katrin Isbruch

**Affiliations:** 1 Department of Psychiatry, Psychotherapy and Psychosomatic Medicine, Evangelisches Krankenhaus Castrop-Rauxel, Academic Teaching Hospital of the University of Duisburg/Essen, Grutholzallee 21, 44577 Castrop-Rauxel, Germany; 2 Department of Psychiatry and Psychotherapy, University of Duisburg/Essen, Virchowstr 174, 45147 Essen, Germany

Unfortunately, the original version of this article [1] contained an error. The author’s name Katrin Isbruch was included twice in the author list. The correct author list has been corrected in the original article and is also included correctly below.

Udo Bonnet^1,2*,^ Claudia Selle^1^, Katrin Isbruch^1^
